# The sexual health of female sex workers compared with other women in England: analysis of cross-sectional data from genitourinary medicine clinics

**DOI:** 10.1136/sextrans-2013-051381

**Published:** 2014-02-03

**Authors:** Louise Mc Grath-Lone, Kimberly Marsh, Gwenda Hughes, Helen Ward

**Affiliations:** 1 Department of Infectious Disease Epidemiology, School of Public Health, Imperial College London, London, UK; 2 Department of HIV & STIs, Centre for Infectious Disease Surveillance and Control, Public Health England, London, UK

**Keywords:** Sexual Health, Surveillance, Prostitution, Women

## Abstract

**Background:**

While female sex workers (FSWs) are assumed to be at increased risk of sexually transmitted infections (STIs), there are limited comparative data with other population groups available. Using routine STI surveillance data, we investigated differences in sexual health between FSWs and other female attendees at genitourinary medicine (GUM) clinics in England.

**Methods:**

Demographic characteristics, STI prevalence and service usage among FSWs and other attendees in 2011 were compared using logistic regression.

**Results:**

In 2011, 2704 FSWs made 8411 recorded visits to 131/208 GUM clinics, (primarily large, FSW-specialist centres in London). FSWs used a variety of services, however, 10% did not have an STI/HIV test at presentation. By comparison with other female attendees, FSWs travelled further for their care and had increased risk of certain STIs (eg, gonorrhoea OR_adj_: 2.76, 95% CI 2.16 to 3.54, p<0.001). Migrant FSWs had better sexual health outcomes than UK-born FSWs (eg, period prevalence of chlamydia among those tested: 8.5% vs 13.5%, p<0.001) but were more likely to experience non-STI outcomes (eg, pelvic inflammatory disease OR_adj_: 2.92, 95% CI 1.57 to 5.41, p<0.001).

**Conclusions:**

FSWs in England have access to high-quality care through the GUM clinic network, but there is evidence of geographical inequality in access to these services. A minority do not appear to access STI/HIV testing through clinics, and some STIs are more prevalent among FSWs than other female attendees. Targeted interventions aimed at improving uptake of testing in FSWs should be developed, and need to be culturally sensitive to the needs of this predominantly migrant population.

## Introduction

Factors associated with sex work (eg, multiple sexual partners, violence and drug use) pose a risk to the health of female sex workers (FSWs).^1^ In a number of countries the prevalence of HIV and sexually transmitted infections (STIs) are higher among FSWs than other women,^2–4^ and some subpopulations, such as migrant FSWs, appear to have worse sexual health outcomes.^5^ In England, FSWs are thought to be at increased risk of STI^6–8^ and to experience barriers to accessing prevention and treatment services.^9–11^ Disparities in sexual health outcomes and service usage between migrant and UK-born FSWs have also been described.^12^ However, much of what is reported is based on information gathered from special studies which are usually small and reliant on self-reported information.

The Genitourinary Medicine Clinic Activity Dataset (GUMCAD) is a patient-level, electronic dataset including diagnoses made and services provided at all genitourinary medicine (GUM) clinics in England that enables analysis of associations between patients’ demographic characteristics and their use of sexual health services and sexual health outcomes. Since 2011, it has included information on whether patients are sex workers (SWs)^13^ presenting a unique opportunity to undertake a comprehensive analysis of SWs seeking sexual healthcare in England using routine national surveillance system data. For the first time, we compare the sexual health outcomes and service usage of FSWs with those of other females attending GUM clinics in England in order to better determine the demographic and clinical characteristics of FSWs, their risk of STI acquisition and patterns of service access. Such information could be used to inform the development of sexual health services better tailored to the needs of this population.

## Methods

### Source of data

Guidelines for collecting and reporting GUMCAD data have previously been published.^14^ Briefly, demographic data is self-reported by patients at first attendance (ie, gender, age, ethnicity, country of birth and postcode of residence) or during a clinical consultation (ie, sexual orientation). (It should be noted that self-reported sexual orientation may not always be congruent with sexual practice,^15^ ie, FSWs may engage in sex with men but identify as homosexual). For each consultation, relevant services and diagnoses are recorded for a patient using a set of uniform codes known as Sexual Health and HIV Activity Property Type (SHHAPT) codes. The SHHAPT code ‘SW’ is attached to visits made by SWs but is not permanently attached to a patient's clinic record; it is recorded independently at each attendance, thereby requiring active coding by healthcare workers. There are no guidelines provided to GUM clinics on how to define sex work or ascertain patients’ SW status, clinics may rely on self-disclosure by patients or on healthcare staff actively asking all or selected patients. A subset of GUMCAD data containing all visits by females between 1 January and 31 December 2011 was extracted from the GUMCAD database held by Public Health England ((PHE), formerly the Health Protection Agency). This subset contained a record of tests, services and diagnoses across attendances within a clinic for each woman. Women recorded as SWs (by the application of the SHAPPT code ‘SW’ during one visit in 2011) were classified as such for any other visits that year. This was to ensure that visits without the ‘SW’ code attached, either due to inconsistent disclosure by FSWs^10^ ^11^ ^16^ or coding errors by staff, were not excluded from the analysis.

### Data analysis

The demographic characteristics of attendees, number of clinic visits and the following service use variables were compared using Pearson χ^2^ tests: the proportion of patients receiving postexposure prophylaxis for HIV following sexual exposure (PEPSE), contraception, STI testing or cervical cytology on any visit in the year and the proportion receiving HIV testing or hepatitis B vaccination where appropriate (ie, excluding individuals who were hepatitis B immune, HIV positive, or recently tested for HIV). Travel for care was investigated by comparing the proportion of patients attending clinics outside their area of residence. Differences in sexual health between FSWs and other female attendees, and between migrant and UK-born FSWs, were assessed by comparing the period prevalence of STIs (ie, chlamydia, gonorrhoea, syphilis, HIV, herpes, genital warts, hepatitis B, hepatitis C, pelvic inflammatory disease (PID), trichomoniasis, scabies, molluscum contagiosum) and other conditions (ie, bacterial vaginosis (BV), candidosis, urinary tract infections (UTI), abnormal cervical cell cytology). Period prevalence was defined as the proportion of individuals tested for an STI in 2011 who experienced an episode of that STI. Patients with SHHAPT codes for chlamydia, gonorrhoea, syphilis and HIV tests were included in the denominator for estimating period prevalence of these infections. As there are no SHAPPT codes to describe the tests, or investigations used to diagnose other STIs/conditions, all individuals were included in the denominator.

Univariate associations between SW status and diagnoses and demographic factors (eg, age, ethnicity, sexual orientation, location, deprivation and migrant status) were investigated using logistic regression. The 2010 index of multiple deprivation score for a patient's postcode of residence was used as a measure of deprivation, and non-UK born FSWs were defined as migrants. Factors with p value<0.10 were included in multivariate logistic regression to explore the effect of SW status on infection and reinfection, adjusting for potential confounders. Women experiencing a second infection with an STI more than 6 weeks after their original diagnosis was recorded as reinfections. Subsequent reinfections were not included in the analysis. This analysis was repeated to compare UK-born and migrant FSW in terms of their demographic characteristics, attendance patterns, service use and STI prevalence and reinfection. Stata V.12 was used for all analyses.

## Results

### Demographic characteristics

In 2011, there were 699 645 women who attended GUM clinics in England, of whom 2704 (0.4%) were identified as SWs. Table 1 shows the demographic characteristics and services used for FSWs and other female attendees. A greater proportion of FSWs than other female attendees were migrants, and FSWs tended to be older. Table 2 shows the demographic characteristics and services used for UK-born and migrant FSWs. There were 98 countries of origin recorded for migrant FSWs, with 46.7% coming from Eastern Europe (51.8% of these were from Romania), 26.1% from South America (94.0% from Brazil) and 16.0% from Asia (42.9% from China). Migrant FSWs were younger than UK-born FSWs.

**Table 1:** Demographic characteristics of and use of services by females attending GUM clinics in England in 2011 by sex worker status

	Female sex workers		Other female attendees		
	(n=2704)		(n=696 941)		
Demographic characteristics	n	%	n	%	p Value
Age*					
<19	118	*4.4*	128 781	*18.5*	
20–24	681	*25.2*	210 452	*30.2*	
25–29	659	*24.4*	135 951	*19.5*	
30–34	483	*17.9*	81 062	*11.6*	
35–44	529	*19.6*	88 752	*12.7*	
45+	228	*8.4*	51 865	*7.4*	**<0.001**
Median	29 years		25 years		**<0.001**
Ethnicity†					
White	1695	*62.7*	508 377	*72.9*	
Mixed	129	*4.8*	23 972	*3.4*	
Asian or Asian British	99	*3.7*	24 523	*3.5*	
Black or Black British	155	*5.7*	74 029	*10.6*	
Other	346	*12.8*	16 860	*2.4*	**<0.001**
Sexual orientation‡					
Heterosexual	2495	*92.3*	607 332	*87.1*	
Homosexual	57	*2.1*	9 314	*1.3*	
Bisexual	56	*2.1*	3 023	*0.4*	**<0.001**
Migrant status§					
UK-born	858	*31.7*	527 065	*75.6*	
Migrant	1666	*61.6*	124 663	*17.9*	**<0.001**
Services used					
Sexual health screen	2424	*89.7*	467 676	*67.1*	**<0.001**
HIV test¶	2405	*89.5*	443 740	*64.4*	**<0.001**
Contraception	878	*32.5*	73 702	*10.6*	**<0.001**
Smear test	337	*12.5*	10 520	*1.5*	**<0.001**
Vaccination (hepatitis B)**	853	*31.9*	6 595	*1.0*	**<0.001**
PEPSE	19	*0.7*	723	*0.1*	**<0.001**

Significant differences (p Value <0.05) highlighted in bold.

*Age was unknown for 6 female sex workers and 78 other female attendees.

†Ethnicity was unknown for 280 female sex workers and 49 180 other female attendees.

‡Sexual orientation was unknown for 96 female sex workers and 45 213 other female attendees.

§Migrant status was unknown for 180 female sex workers and 45 213 other female attendees.

¶Excluding individuals who were known HIV positive, or where a HIV test was not appropriate (n/2686 for female sex workers and n/689 254 for other female attendees).

**Excluding individuals who were hepatitis B immune (n/2676 for female sex workers and n/696 265 for other female attendees).

GUM, genitourinary medicine; PEPSE, postexposure prophylaxis for HIV following sexual exposure.

**Table 2:** Demographic characteristics of and use of services by female sex workers attending GUM clinics in England in 2011 by migrant status

	UK-born FSW		Migrant FSW		
	(n=858)		(n=1666)		
Demographic characteristics	n	%	n	%	p Value
Age*					
<19	47	*5.5*	68	*4.1*	
20–24	211	*24.6*	442	*26.5*	
25–29	178	*20.7*	431	*25.9*	
30–34	113	*13.2*	329	*19.8*	
35–44	182	*21.2*	317	*19.0*	
45+	124	*14.5*	77	*4.6*	**<0.001**
Median	29 years		28 years		**0.01**
Ethnicity†					
White	616	*71.8*	1021	*61.3*	
Mixed	35	*4.1*	92	*5.5*	
Asian or Asian British	23	*2.7*	69	*4.1*	
Black or Black British	74	*8.6*	75	*4.5*	
Other	10	*1.2*	315	*18.9*	**<0.001**
Sexual orientation‡					
Heterosexual	742	*86.5*	1576	*94.6*	
Homosexual	30	*3.5*	21	*1.3*	
Bisexual	32	*3.7*	18	*1.1*	**<0.001**
U.K.	858	*100.0*			
Europe§			84	*5.0*	
Eastern Europe			778	*46.7*	
Africa			73	*4.4*	
Asia			266	*16.0*	
Australia			9	*0.5*	
North America			21	*1.3*	
South America			435	*26.1*	n/a
Sexual health screen	789	*92.0*	1576	*94.6*	**0.01**
HIV test¶	724	*87.4*	1511	*97.2*	**<0.001**
Contraception	156	*18.2*	685	*41.1*	**<0.001**
Smear test	51	*5.9*	269	*16.2*	**<0.001**
Vaccination (hepatitis B)**	228	*27.9*	570	*36.6*	**<0.001**
PEPSE	7	*0.8*	10	*0.6*	0.53

Significant differences (p Value <0.05) highlighted in bold.

*Age was unknown for 3 UK-born and 2 migrant female sex workers.

†Ethnicity was unknown for 100 UK-born and 94 migrant female sex workers.

‡Sexual orientation was unknown for 54 UK-born and 51 migrant female sex workers.

§Europe excludes UK and Eastern Europe.

¶Excluding individuals who were known HIV positive, or where a HIV test was not appropriate (n/828 for UK-born female sex workers and n/1554 for migrant female sex workers).

**Excluding individuals who were hepatitis B immune (n/816 for UK-born female sex workers and n/1557 for migrant female sex workers).

FSW, female sex worker; GUM, genitourinary medicine; PEPSE, postexposure prophylaxis for HIV following sexual exposure.

### Attendance patterns

The 2704 FSWs identified in this study made 8411 visits to 131 of the total 208 GUM clinics in 2011. They made more visits than other female attendees (mean number of visits in 2011; 3.1 vs 1.7, p<0.001) and were more likely to have had a repeat visit (71.2% vs 35.6%, p<0.001). Migrant FSWs made more visits than UK-born FSWs (mean number of visits in 2011; 3.7 vs 2.9, p<0.001). Visits by FSWs were geographically concentrated; they were more likely to attend a clinic in London than other attendees (74.7% vs 28.4%, p<0.001). They were also more likely to attend London clinics even when residing elsewhere; 14.5% of FSW and 6.6% of other female attendees attending clinics in London were not London residents (p<0.001). Visits by FSWs were concentrated in large clinics providing SW-specific services; more than three-quarters (79.4%) of their visits were recorded at just 12 clinics, seven of which were in London. These 12 clinics accounted for 92.2% of migrant FSW visits compared with 57.8% of UK-born FSWs.

### Service use

FSWs were more likely to use non-testing services such as contraception, smear tests and hepatitis B vaccination than other attendees (table 1), and a greater proportion of FSWs had a HIV test or sexual health screen (which tests for chlamydia, gonorrhoea and syphilis). As FSW visits were not equally distributed across all clinics we investigated whether the higher proportion of FSWs tested may be due to higher levels of testing overall at the 131 clinics they attended. However, the significant association remained when the analysis was restricted to these clinics (88.9% vs 63.5%, p<0.001 for sexual health screens; 87.8% vs 61.4%, p<0.001 for HIV tests). Migrant FSWs were more likely than UK-born FSWs to have a sexual health screen or HIV test, and to use non-testing services, with the exception of PEPSE (table 2).

### Sexual health

There were no significant differences in the period prevalence of HIV or syphilis between FSWs and other female attendees. Chlamydia was the most prevalent STI diagnosed in both groups with a significantly higher period prevalence among FSWs (table 3). FSWs were almost twice as likely to be diagnosed with chlamydia, and three times more likely to be diagnosed with gonorrhoea than other female attendees, adjusting for demographic factors. As FSWs made more visits on average than other female attendees, this increased prevalence may have been linked to the higher number of opportunities FSWs had to be diagnosed. As our model did not adjust for number of visits, we also compared prevalence of STI diagnosis in those tested at first visit and found that FSWs were still twice as likely to be diagnosed with chlamydia (2.9% vs 1.3%, p<0.001) and four times more likely to be diagnosed with gonorrhoea (0.8% vs 0.2%, p<0.001) than other female attendees. Other conditions such as hepatitis B, hepatitis C, scabies, BV, candidosis, PID, UTI and abnormal cervical cytology were also higher among FSWs, though other female attendees had a higher prevalence of genital warts. While reinfections with some STIs were more common in FSWs than in other females, the only statistically significant differences were for chlamydia reinfections (6.6% of infected FSWs became reinfected in 2011 vs 3.4% of other attendees, p=0.02) and BV recurrences (7.4% vs 3.7%, p=0.004). UK-born FSWs had a higher prevalence of chlamydia and trichomoniasis than migrant FSWs (table 4); however, the prevalence of candidosis, UTI, PID and abnormal cervical cytology were higher among UK-born FSWs. Adjusting for demographic factors and other diagnoses, only differences in PID and trichomoniasis prevalence remained statistically significant. Migrant FSWs were 83% less likely to be diagnosed with trichomoniasis than UK-born FSWs but three times more likely to be diagnosed with PID. There were no statistically significant differences in reinfections between migrant and UK-born FSWs.

**Table 3:** Period prevalence of selected STIs and other conditions among females attending GUM clinics in England in 2011 by sex worker status

	Female sex workers		Other female attendees		Association with being a female sex worker					
	Period prevalence (%)	Diagnosed/tested* (n/N)	Period prevalence (%)	Diagnosed/tested* (n/N)	OR (unadjusted)	95% CI	p Value	OR (adjusted†)	95% CI	p Value
Chlamydia‡	10.1	*257/2534*	8.5	*47 336/556 686*	1.28	1.12 to 1.46	**<0.001**	1.93	1.61 to 2.33	**<0.001**
Gonorrhoea	2.7	*68/2534*	1.0	*5756/553 960*	2.76	2.16 to 3.54	**<0.001**	2.75	1.87 to 4.04	**<0.001**
Syphilis	0.1	*3/2380*	0.1	*285/466 248*	2.97	0.95 to 9.33	0.06	2.55	0.59 to 11.03	0.21
HIV§	0.2	*5/2405*	0.2	*895/443 740*	1.16	0.48 to 2.80	0.74	0.74	0.18 to 3.04	0.68
Herpes	2.3	*62/2704*	2.8	*19 165/696 941*	0.83	0.65 to 1.07	0.15	0.91	0.63 to 1.32	0.63
Genital warts	2.8	*76/2704*	5.0	*34 676/696 941*	0.55	0.44 to 0.69	**<0.001**	1.07	0.79 to 1.45	0.68
Hepatitis B	0.6	*16/2704*	0.1	*429/696 941*	9.66	5.86 to 15.94	**<0.001**	1.63	0.51 to 5.24	0.82
Hepatitis C	0.2	*6/2704*	0.04	*260/696 941*	5.96	2.65 to 13.40	**<0.001**	1.68	0.39 to 7.27	0.49
Trichomoniasis	0.9	*24/2704*	0.8	*5459/696 941*	1.13	0.76 to 1.70	0.54	1.64	0.96 to 2.82	0.07
Scabies	0.1	*3/2704*	0.03	*207/696 941*	3.74	1.20 to 11.69	**0.02**	8.18	1.81 to 36.89	**0.01**
Molluscum contagiosum	0.5	*14/2704*	0.5	*3585/696 941*	1.01	0.60 to 1.70	0.98	1.51	0.79 to 2.86	0.21
Bacterial vaginosis	25.6	*691/2704*	12.7	*88 238/696 941*	2.37	2.17 to 2.58	**<0.001**	2.30	2.04 to 2.59	**<0.001**
Candidosis	16.6	*450/2704*	7.5	*52 612/696 941*	2.45	2.21 to 2.71	**<0.001**	1.92	1.68 to 2.19	**<0.001**
Urinary tract infection	7.8	*212/2704*	2.2	*14 984/696 941*	3.87	3.36 to 4.46	**<0.001**	2.29	1.90 to 2.75	**<0.001**
Pelvic inflammatory disease	10.6	*286/2704*	2.3	*16 204/696 941*	4.97	4.39 to 5.62	**<0.001**	4.21	3.58 to 4.95	**<0.001**
Other conditions	19.6	*530/2704*	9.2	*64 402/696 941*	1.77	1.61 to 1.95	**<0.001**	1.37	1.20 to 1.56	**<0.001**
Abnormal cervical cell cytology	32.9	*111/337*	16.3	*1716/10 520*	17.34	14.26 to 21.09	**<0.001**	8.66	6.60 to 11.37	**<0.001**

Significant differences (p Value <0.05) highlighted in bold.

*For chlamydia, gonorrhoea, syphilis and HIV, N=women with a recorded test for the STI of interest; for abnormal cell cytology, N=women with a recorded smear test; and for all other STIs/conditions without a SHAPPT code for the relevant diagnostic test/investigation, N=all women.

†Adjusting for age, ethnicity, migrant status, sexual orientation, location (inner London, outer London or outside London), deprivation of postcode of residence and chlamydia and/or gonorrhoea diagnosis.

‡Code suffixes to identify oral or rectal chlamydia infections were introduced to GUMCAD in 2011. However, as the use of these suffixes was not consistent across all clinics in 2011, the data presented here includes all chlamydia infections. In future, it will be possible to provide information on the site of infection.

§New HIV diagnoses in 2011.

GUM, genitourinary medicine; GUMCAD, Genitourinary Medicine Clinic Activity Dataset; STI, sexually transmitted infection.

**Table 4:** Period prevalence of selected STIs and other conditions among female sex workers attending GUM clinics in England in 2011 by migrant status

	UK-born FSW		Migrant FSW		Association with being a migrant female sex worker					
	Period prevalence (%)	Diagnosed/tested* *n/N*	Period prevalence (%)	Diagnosed/tested* *n/N*	OR (unadjusted)	95% CI	p Value	OR (adjusted†)	95% CI	p Value
Chlamydia‡	*13.5*	*108/799*	*8.5*	*134/1581*	0.59	0.46 to 0.79	**<0.001**	0.61	0.38 to 0.97	**0.04**
Gonorrhoea	*3.2*	*25/792*	*2.4*	*37/1576*	0.74	0.44 to 1.23	0.25	1.00	0.38 to 2.70	0.99
Syphilis	*0.1*	*1/722*	*0.1*	*2/1511*	0.96	0.09 to 10.56	0.97	0.17	0.01 to 4.53	0.29
HIV§	*0.0*	*0/724*	*0.3*	*4/1511*	Omitted			Omitted		
Herpes	*3.0*	*26/858*	*2.1*	*35/1666*	0.69	0.41 to 1.15	0.15	0.59	0.21 to 1.68	0.32
Genital warts	*2.8*	*24/858*	*2.9*	*49/1666*	1.05	0.64 to 1.73	0.84	1.92	0.72 to 5.10	0.19
Hepatitis B	*0.1*	*1/858*	*0.7*	*12/1666*	6.22	0.81 to 47.90	0.08	Omitted		
Hepatitis C	*0.5*	*4/858*	*0.1*	*2/1666*	0.26	0.05 to 1.40	0.12	Omitted		
Trichomoniasis	*1.5*	*13/858*	*0.5*	*9/1666*	0.35	0.15 to 0.83	**0.02**	0.17	0.05 to 0.59	**0.01**
Scabies	*0.1*	*1/858*	*0.1*	*2/1666*	1.03	0.09 to 11.38	0.98	0.42	0.02 to 11.98	0.62
Molluscum contagiosum	*0.3*	*3/858*	*0.6*	*10/1666*	1.72	0.47 to 6.27	0.41	0.77	0.12 to 5.37	0.80
Bacterial vaginosis	*26.3*	*226/858*	*26.2*	*436/1666*	0.99	0.82 to 1.20	0.93	0.81	0.58 to 1.15	0.24
Candidosis	*13.3*	*114/858*	*18.9*	*315/1666*	1.52	1.21 to 1.92	**<0.001**	1.29	0.87 to 1.92	0.21
Urinary tract infection	*4.8*	*41/858*	*9.3*	*155/1666*	2.04	1.43 to 2.91	**<0.001**	1.34	0.77 to 2.32	0.30
Pelvic inflammatory disease	*3.8*	*33/858*	*14.3*	*238/1666*	4.17	2.87 to 6.06	**<0.001**	2.92	1.57 to 5.41	**0.001**
Other conditions	*20.5*	*176/858*	*19.6*	*327/1666*	0.95	0.77 to 1.16	0.60	0.83	0.57 to 1.19	0.30
Abnormal cervical cell cytology	*25.5*	*13/51*	*35.3*	*95/269*	3.93	2.19 to 7.06	**<0.001**	1.19	0.52 to 2.73	0.69

Significant differences (p Value <0.05) highlighted in bold.

*For chlamydia, gonorrhoea, syphilis and HIV, N=women with a recorded test for the STI of interest; for abnormal cell cytology, N=women with a recorded smear test; and for all other STIs/conditions without a SHAPPT code for the relevant diagnostic test/investigation, N=all women.

†Adjusting for age, ethnicity, sexual orientation, location (inner London, outer London or outside London), deprivation of postcode of residence and chlamydia and/or gonorrhoea diagnosis.

‡Code suffixes to identify oral or rectal chlamydia infections were introduced to GUMCAD in 2011. However, as the use of these suffixes was not consistent across all clinics in 2011, the data presented here includes all chlamydia infections. In future, it will be possible to provide information on the site of infection.

§New HIV diagnoses in 2011.

FSW, female sex worker: GUM, genitourinary medicine; GUMCAD, Genitourinary Medicine Clinic Activity Dataset; STI, sexually transmitted infection.

## Discussion

For the first time, we have been able to provide a comprehensive description of the characteristics and sexual health needs of FSWs receiving care at GUM clinics in England. FSWs appear to be at higher risk of infection and reinfection with certain STIs than other female attendees; however, the levels of STI among FSWs reported here are lower than in comparable studies. While services, such as STI testing, vaccination and contraception are more frequently accessed by FSWs than other attendees, there is evidence of missed opportunities.

One limitation of our study is the likely underestimation of the number of FSWs attending GUM clinics, partly due to under-disclosure by FSWs but also because of an absence of guidelines on how SW status should be ascertained, or lack of coding by staff. Additionally, FSWs attending GUM clinics may not be representative of the wider FSW population, as those who attend GUM clinics for testing and treatment may have different risk behaviours than those who do not. Lack of knowledge about the different health-seeking behaviours among FSWs limits the generalisability of the conclusions that can be drawn from this analysis to FSWs who attend GUM clinics. Nonetheless, by comparison with special SW studies, our study provides a national picture of the sexual health of FSWs attending GUM clinics and their use of services, enabling geographic variations in service access to be explored and specific sexual health needs of FSWs to be investigated.

In our study, FSWs were older, a greater proportion lived in London and, most notably, three times as many were migrants compared with other attendees. Sociodemographic differences such as these may be useful for tailoring interventions and services for FSWs, for example, deciding on the location of outreach services and deciding which languages to offer them in. Country of origin of migrant FSWs also has implications for service priorities at clinics, for example, ensuring HIV testing is available to the large number from Brazil where HIV prevalence among FSWs is 6.2%.^17^ Visits by FSWs were not equally distributed across clinics, only 131/208 reported an attendance by FSWs in 2011, and large, specialist centres with SW-specific services appear to play a crucial role in their sexual healthcare provision. One clinic in London reported almost a quarter of all FSW visits, with some FSWs who attended this clinic living as much as 300 miles away in North West England. FSWs living outside of London were more likely to attend services in London than other female attendees which may be a reflection of FSWs working patterns. FSWs working in London but living elsewhere in the country may choose to attend a clinic near their workplace rather than their area of residence. However, the pattern of visiting clinics in London when living elsewhere, the concentration of visits at large, specialist centres and the low number, or absence, of FSW attendances in some areas of the country, could also signify geographical inequalities in terms of access to, or awareness of, suitable services for FSWs. For example, in Cumbria, there were no attendances by FSWs reported to our surveillance system in 2011 even though a study mapping the sex market in the region identified more than 180 male and female SWs.^18^ It is also possible that FSWs are accessing these clinics, but the SHAPPT code ‘SW’ is not being applied to their consultation as they do not disclose, or are not asked about, their involvement in sex work. Using routine surveillance data to identify clinics that are frequently and rarely visited by SWs may be useful for commissioners and service planners to investigate areas of potential unmet needs, and to strategically plan services and outreach programmes.

While FSWs attending GUM clinics were significantly more likely than other attendees to have a sexual health screen or HIV test, over 10% of FSWs were not tested. These FSWs may have been assessed not to need testing when they attended, or may access testing through primary health care providers,^11^ which do not report to our surveillance system; however, it is likely that there remains a minority of FSWs who are not testing for STIs at all, as has been seen in other UK studies.^19^ ^20^ That one in eight FSWs attending a GUM clinic had no sexual health screen or HIV test despite being already engaged with healthcare services, represents major missed opportunities. Studies have suggested that migrant FSWs have poorer access and are less likely to engage with services than UK-born FSWs,^12^ perhaps due to lack of knowledge or fears related to their legal/immigration status.^21^ ^22^ However, in our study, migrant FSWs made more visits on average than UK-born FSWs, and a greater proportion had a sexual health screen or HIV test. A greater proportion of migrant FSWs also used other services, such as contraception and cervical cytology, which highlights the vital role GUM clinics play in meeting their broader sexual and reproductive healthcare needs, but may also indicate unmet need for primary care-type services, similar to that expressed by FSWs in Bristol.^11^ The higher prevalence of non-sexually transmitted illness (eg, UTI) and the increased risk of PID further support this theory. This may have implications for service planning and intervention design for FSWs which may need to adopt an integrated approach to improving sexual health in the wider context of improving their general health.

FSWs have been shown to be at greater risk of STI when compared with the general population, for example, STIs were 9–60 times more common in street-based FSWs than in females aged 16–44 years from the General Household Survey (GHS).^19^ In our study, FSWs were twice as likely as other female attendees to be diagnosed with chlamydia and almost three times more likely to be diagnosed with gonorrhoea, but there was no significant difference in the period prevalence of HIV or syphilis. Our calculated odds of infection for FSWs by comparison with other females may be lower than those reported elsewhere, as our comparative population of GUM clinic attendees is likely to be at higher risk than the GHS population. Furthermore, street-based FSWs are a higher risk group than other FSWs^10^ ^23–25^ and are not likely to be representative of FSWs attending GUM clinics. The increased risk of certain STIs among FSWs that we identified may not be a consequence of sex work per se, but rather of other factors associated with sex work. STI acquisition among FSWs has been shown to be associated with intravenous drug use (IDU) and the use of condoms with, and number of, non-paying casual partners^6^ ^24^ rather than the number of clients or duration of sex work. As our surveillance system does not gather data on sexual and drug-injecting behaviours, the influence of these factors on STI risk could not be determined. Currently, the collection of behavioural data (including IDU) through GUMCAD is being piloted by PHE, and so the impact of these factors may be explored in the future. More than 10% of FSWs infected with chlamydia and more than 30% diagnosed with BV in 2011 experienced a reinfection or recurrence during the year, suggesting opportunities exist for improving clinic-based interventions to prevent repeated risk exposure.

Migrant FSWs were less likely to be diagnosed with chlamydia or trichomoniasis than UK-born FSWs, but there were no significant differences for infection with other STIs between the groups. While our findings contradict those of other studies in the UK^7^ ^12^ that suggest migrant FSWs are at higher risk of STI than indigenous FSWs, they must be interpreted cautiously given the limitation in generalisability of this study to FSWs attending GUM clinics. Previous studies reporting poorer sexual health outcomes among migrant FSWs have also shown they have poorer access to services such as GPs,^12^ thus, the comparatively better sexual health outcomes observed among the migrant FSWs in our study may be a reflection of their increased use of services. The differences in their working conditions could also be a contributing factor; it is thought migrant FSWs in England work almost exclusively indoors,^12^ ^26^ a lower-risk occupational environment,^23^ ^25^ however, this hypothesis was not tested in this study as this type of information is not gathered routinely through GUMCAD. Understanding the cause of the variation in sexual health between migrant and UK-born FSWs in the future will be important for effective intervention design.

Overall, FSWs in England have access to high-quality sexual health care through the GUM clinic network, but there is evidence of geographical inequality in access to these services. Though uptake is high, a minority of FSWs do not appear to access STI/HIV testing through clinics, and further quantitative and qualitative studies to identify the barriers to testing among FSWs are warranted. Some STIs are more prevalent among FSWs than other female attendees, and the high levels of reinfection with chlamydia and BV experienced by FSWs should be investigated further. Targeted interventions aimed at improving uptake of testing and reducing the risk of repeat infection in this population should be developed, and need to be culturally sensitive to the needs of this predominantly migrant population.

**Key messages**

- FSWs appear to be at higher risk of certain sexually transmitted infections and reinfections compared with other female genitourinary medicine (GUM) clinic attendees, even adjusting for demographic factors.
- FSWs have access to high-quality sexual healthcare through the GUM clinic network, but there is evidence of geographical inequality in access to services.
- Differences in service usage among FSWs by migrant status may indicate migrant FSWs experience unmet needs for primary care-type services.
- There is little variation in sexual health outcomes among FSWs by migrant status; however, chlamydia infection is more common in UK-born FSWs.

## References

[1] Spice W Management of sex workers and other high-risk groups. Occup Med-Oxford 2007;57:322–8 10.1093/occmed/kqm045 17656497

[2] Scott G Peacock W Cameron S Outreach STD clinics for prostitutes in Edinburgh. Int J STD AIDS 1995;6:197–200 7647123 10.1177/095646249500600310

[3] Mak RP Van Renterghem L Traen A Chlamydia trachomatis in female sex workers in Belgium: 1998–2003. Sex Transm Infect 2005;81:89–90 15681731 10.1136/sti.2004.010272 PMC1763731

[4] Zermiani M Mengoli C Rimondo C Prevalence of sexually transmitted diseases and hepatitis C in a survey of female sex workers in the north-East of Italy. Open AIDS J 2012;6:60–4 22833775 10.2174/1874613601206010060 PMC3401890

[5] Platt L Grenfell P Fletcher A Systematic review examining differences in HIV, sexually transmitted infections and health-related harms between migrant and non-migrant female sex workers. Sex Transm Infect 2013;89:311–19 23112339 10.1136/sextrans-2012-050491

[6] Ward H Day S Mezzone J Prostitution and risk of HIV: female prostitutes in London. Brit Med J 1993;307:356–8 8374417 10.1136/bmj.307.6900.356 PMC1678221

[7] Fox J Taylor GP Day S How safe is safer sex? High levels of HSV-1 and HSV-2 in female sex workers in London. Epidemiol Infect 2006;134:1114–19 16569273 10.1017/S0950268806006133 PMC2870498

[8] Creighton S Tariq S Perry G Sexually transmitted infections among UK street-based sex workers. Sex Transm Infect 2008;84:32–3 17901086 10.1136/sti.2007.026443

[9] Bell G Rogstad K Off-street sex workers and their use of genitourinary medicine services. Int J STD AIDS 2000;11:592–3 10997502 10.1258/0956462001916588

[10] Ibbitson M Out of the sauna: sexual health promotion with ‘off street’ sex workers. J Epidemiol Commun H 2002;56:903–4 10.1136/jech.56.12.903 PMC1757006 12461109

[11] Jeal N Salisbury C Self-reported experiences of health services among female street-based prostitutes: a cross-sectional survey. Brit J Gen Pr 2004;54:515–19 PMC1324803 15239913

[12] Platt L Grenfell P Bonell C Risk of sexually transmitted infections and violence among indoor-working female sex workers in London: the effect of migration from Eastern Europe. Sex Transm Infect 2011;87:377–84 21572111 10.1136/sti.2011.049544

[13] Health Protection Agency. Revisions to KC60 (SHHAPT) codes: Specification and rationale. London, UK, 2011:1–20

[14] Health Protection Agency. GUMCAD Genitourinary Medicine Clinic Activity Dataset Guidance to clinic staff. 2011:1–27

[15] Harcourt C Donovan B The many faces of sex work. Sex Transm Infect 2005;81:201–6 15923285 10.1136/sti.2004.012468 PMC1744977

[16] Cohan D Lutnick A Davidson P Sex worker health: San Francisco style. Sex Transm Infect 2006;82:418–22 16854996 10.1136/sti.2006.020628 PMC2563853

[17] Malta M Magnanini MMF Mello MB HIV prevalence among female sex workers, drug users and men who have sex with men in Brazil: a systematic review and meta-analysis. BMC Public Health 2010;10:317 20529289 10.1186/1471-2458-10-317 PMC2898825

[18] Barefoot Research and Evaluation. Study into the extent and characteristics of the sex market and sexual exploitation in Cumbria. Northern Rock Foundation, 2012:1–35

[19] Jeal N Salisbury C A health needs assessment of street-based prostitutes: cross-sectional survey. J Public Heal 2004;26:147–51 10.1093/pubmed/fdh124 15284317

[20] Mironski M Sexual Health Needs Assessment: Commercial Sex Workers (CSW) Hull and East Riding of Yorkshire. Hull and East Riding Sexual and Reproductive Healthcare Partnership, 2010:1–34

[21] TAMPEP. Activity report. 2007:179

[22] UK Network of Sex Work Projects. Good Practice Guidance: Working with Migrant Sex Workers. UK NSWP, 2008:1–32

[23] Jeal N Salisbury C Health needs and service use of parlour-based prostitutes compared with street-based prostitutes: a cross-sectional survey. BJOG 2007;114:875–81 17567420 10.1111/j.1471-0528.2007.01379.x

[24] Ward H Day S Weber J Risky business: health and safety in the sex industry over a 9 year period. Sex Transm Infect 1999;75:340–3 10616360 10.1136/sti.75.5.340 PMC1758230

[25] Jeal N Salisbury C Turner K The multiplicity and interdependency of factors influencing the health of street-based sex workers: a qualitative study. Sex Transm Infect 2008;84:381–5 18596067 10.1136/sti.2008.030841

[26] Home Office. Paying the price: a consultation paper on prostitution. Home Office Communication Directorate: London, UK, 2004

